# Vitamin B6 Acquisition and Metabolism in *Schistosoma mansoni*

**DOI:** 10.3389/fimmu.2020.622162

**Published:** 2021-02-04

**Authors:** Akram A. Da’dara, Manal Elzoheiry, Samar N. El-Beshbishi, Patrick J. Skelly

**Affiliations:** ^1^ Molecular Helminthology Laboratory, Department of Infectious Disease and Global Health, Cummings School of Veterinary Medicine, Tufts University, North Grafton, MA, United States; ^2^ Department of Medical Parasitology, Faculty of Medicine, Mansoura University, Mansoura, Egypt

**Keywords:** pyridoxal phosphate, PLP, schistosome, parasite, ectoenzyme, alkaline phosphatase

## Abstract

Schistosomes are parasitic platyhelminths that currently infect >200 million people globally. The adult worms can live within the vasculature of their hosts for many years where they acquire all nutrients necessary for their survival and growth. In this work we focus on how *Schistosoma mansoni* parasites acquire and metabolize vitamin B6, whose active form is pyridoxal phosphate (PLP). We show here that live intravascular stage parasites (schistosomula and adult males and females) can cleave exogenous PLP to liberate pyridoxal. Of the three characterized nucleotide-metabolizing ectoenzymes expressed at the schistosome surface (SmAP, SmNPP5, and SmATPDase1), only SmAP hydrolyzes PLP. Heat-inactivated recombinant SmAP can no longer cleave PLP. Further, parasites whose SmAP gene has been suppressed by RNAi are significantly impaired in their ability to cleave PLP compared to controls. When schistosomes are incubated in murine plasma, they alter its metabolomic profile—the levels of both pyridoxal and phosphate increase over time, a finding consistent with the action of host-exposed SmAP acting on PLP. We hypothesize that SmAP-mediated dephosphorylation of PLP generates a pool of pyridoxal around the worms that can be conveniently taken in by the parasites to participate in essential, vitamin B6-driven metabolism. In addition, since host PLP‐dependent enzymes play active roles in inflammatory processes, parasite-mediated cleavage of this metabolite may serve to limit parasite-damaging inflammation. In this work we also identified schistosome homologs of enzymes that are involved in intracellular vitamin B6 metabolism. These are pyridoxal kinase (SmPK) as well as pyridoxal phosphate phosphatase (SmPLP-Ph) and pyridox(am)ine 5’-phosphate oxidase (SmPNPO) and cDNAs encoding these three enzymes were cloned and sequenced. The three genes encoding these enzymes all display high relative expression in schistosomula and adult worms suggestive of robust vitamin B6 metabolism in the intravascular life stages.

## Introduction

Schistosomes are intravascular parasitic worms that cause the chronic debilitating disease, schistosomiasis. More than 200 million people are infected with these parasites around the world and >800 million live at risk of infection (1). The disease is a chronic inflammatory disorder that is associated with disabling anemia, growth stunting and poor performance at school and at work. People can become infected after contacting fresh water that contains free-living larval stages (cercariae). After penetrating host skin, cercariae transform into juvenile, intra-mammalian forms called schistosomula. These migrate through the bloodstream to the portal vein, where they mature into adult males and females. The adults pair and couples migrate to the blood vessels around the intestines or the bladder (depending on the species) where egg laying begins.

Schistosomes can live for many years within the vasculature of their hosts where they acquire all nutrients necessary for survival and growth. While the worms have a mouth and a gut, many metabolites are imported into the worms directly across their host-interactive tegument (skin) (2, 3). We have characterized schistosome tegumental proteins that are involved in the uptake and metabolism of amino acids, glucose, and water (4–9). In this work, we focus on parasite acquisition of vitamin B6 and its biochemistry in one of the three major schistosomes of humans—*Schistosoma mansoni.*

Vitamin B6 comprises a group of interconvertible, chemically similar compounds called vitamers. Collectively, six vitamers comprise vitamin B6. The active form of the vitamin is pyridoxal phosphate (PLP) which functions as a coenzyme in a wide variety of enzymatic reactions (over 140 to date) related to amino acid, glucose, and lipid metabolism (10, 11). Vitamin B6 also participates in various non-enzymatic reactions as an antioxidant, carbonyl scavenger and metal chelator (12). The vitamin is critically important for normal growth, cognitive development, and immune function (11). Animals are auxotrophic for vitamin B6 and require it as a supplement; schistosomes must acquire this vital metabolite from their hosts.

Three vitamin B6 vitamers are: pyridoxal (PL), pyridoxine (PN), and pyridoxamine (PM). PL can be directly converted into active vitamin B6 (PLP) by phosphorylation *via* a pyridoxal kinase enzyme (11, 13). This kinase can similarly phosphorylate PN and PM, but an additional enzyme—Pyridox(am)ine 5’-Phosphate Oxidase—is required to convert these metabolites into active vitamin B6 (PLP) (11, 13).

This study was prompted by our work on how schistosomes alter the murine plasma metabolome. When adult worms are incubated in murine plasma the levels of pyridoxal and phosphate increase over time. This observation is most easily understood if the worms have an ability to cleave any PLP that is available in the plasma, thereby generating free pyridoxal and phosphate. Schistosome express three distinct nucleotide-metabolizing ectoenzymes (NMEEs) on their external surface and we hypothesized that one of these mediated PLP cleavage. These enzymes are: *S. mansoni* ATPdiphosphohydrolase1 (SmATPDase1), *S. mansoni* phosphodiesterase/pyrophosphatase (SmNPP5), and *S. mansoni* alkaline phosphatase, (SmAP). All three enzymes have been immunolocalized to the schistosome tegument (skin) and have been shown to be expressed at the schistosome host-interactive surface in tegument proteomic studies (14–16). SmATPDase1 is an ~61 kDa apyrase that can degrade ATP and ADP in a cation dependent manner (17, 18). SmNPP-5 is a ~53 kDa protein with a single C-terminal transmembrane domain that is expressed exclusively in the intra-mammalian life stages and has been shown to cleave ADP and to block platelet aggregation *in vitro* (19–21). SmAP is a ~60 kDa glycosylphosphatidylinositol (GPI)-anchored protein that is expressed in the tegument and in the internal tissues of the intravascular worms (18, 22). SmAP has been shown to cleave several nucleoside monophosphates including AMP, CMP, GMP and TMP (23). SmAP additionally hydrolyzes the bioactive lipid sphingosine-1-phosphate (S1P) (23) as well as the proinflammatory and prothrombotic polymer, polyphosphate (polyP) (24).

In this work, we set out to measure the ability of intravascular schistosomes to hydrolyze extracellular PLP and to determine which (if any) of the three NMEEs listed above is involved. Further, we identified schistosome homologs of enzymes that are involved in vitamin B6 metabolism, cloned their cDNAs and examined their developmental expression profiles. In this manner, the work provides insight into the acquisition and metabolism of an essential nutrient in *Schistosoma mansoni*.

## Materials and Methods

### Parasites and Mice

The Puerto Rican strain of *Schistosoma mansoni* was used. Adult male and female parasites were recovered by perfusion from Swiss Webster mice that were infected with ~100 cercariae, 7 weeks previously (25). Schistosomula were prepared from cercariae released from infected snails. All parasites were cultured in DMEM/F12 medium supplemented with 10% heat-inactivated fetal bovine serum, 200 µg/ml streptomycin, 200 U/ml penicillin, 1 µM serotonin, 0.2 µM Triiodo-l-thyronine, 8 µg/ml human insulin, and were maintained at 37°C, in an atmosphere of 5% CO_2_ (23). Parasite eggs were isolated from infected mouse liver tissue (25). All protocols involving animals were approved by the Institutional Animal Care and Use Committees (IACUC) of Tufts University.

### Pyridoxal and Phosphate Detection in Murine Plasma

Blood was collected from the tail veins of 10 Swiss Webster mice into heparinized tubes. Blood cells were pelleted by brief centrifugation and the plasma generated was pooled and aliquoted. Adult schistosomes (~50 pairs) were incubated in one 500 µl murine plasma aliquot which was incubated at 37°C. A control aliquot (without worms) was similarly treated. Samples, collected at baseline (0 min) and after 20- and 60-min incubation with or without parasites, were subjected to metabolomic analysis at Metabolon Inc. The relative levels of pyridoxal and phosphate are described; these are extracted from a global metabolomics analysis carried out using the pipeline developed by Metabolon. At least four samples per treatment/time point were tested. Briefly, each plasma sample was prepared by solvent extraction and the resulting extract was split into equal parts and then applied to gas chromatography/mass spectrometry (GC/MS) and liquid chromatography tandem MS (LC/MS/MS) platforms (26). Pyridoxal and phosphate were each identified by their retention time and mass by comparison to purified standards. Results are expressed relative to the baseline measurement (0 min), set at 1.

### PLP Hydrolysis by Living Parasites and by Recombinant Schistosome Enzymes

To monitor PLP hydrolysis by living schistosomes, schistosomula (in groups of ~2,000) or adult male or female worms (either singly or in groups of 2 or 3) were incubated in 200 µl assay buffer (50 mM Tris-HCl buffer (pH 9), 5 mM KCl, 135 mM NaCl, 5 mM KCl, 10 mM glucose, 10 mM MgCl_2_) containing 2 mM PLP (Sigma Aldrich). Aliquots (25 µl) were recovered at selected time points and any phosphate generated following PLP cleavage was measured using a Phosphate Colorimetric Assay Kit (BioVision), following the manufacturer’s instructions.

Recombinant SmAP, SmNPP5, and SmATPDase1 enzymes, expressed in CHO-S cells, were prepared as described (17, 21, 23) and each was assessed for its ability to cleave PLP in the assay described above. For both SmAP and SmNPP5, 0.5 µg protein was tested in each 100 µl reaction. Since SmATPDase1 is only active when expressed on the surface of CHO cells (17), PLP substrate was added to 10^5^ cells expressing SmATPDase1 or control cells not expressing any heterologous protein. At selected time points, aliquots were recovered from all experimental conditions and any released inorganic phosphate was measured using the Phosphate Colorimetric Assay Kit, as above. As a negative control, in some experiments recombinant proteins were heat treated at 95°C for 10 min before being included in activity assays.

### Gene Suppression Using RNAi

Adult worms were electroporated with either an siRNA (10 µg) targeting SmAP (SmAP siRNA 1: 5’-AAGAAATCAGCAGATGAGAGATTTAAT-3’), or with a control siRNA that targeted no sequence in the schistosome genome (Control: 5’-CTTCCTCTCTTTCTCTCCCTTGTGA-3’) or with no siRNA, as described previously (22). To assess the level of target gene suppression, RNA was isolated from some worms from each group two days later using the TRIzol Reagent (Thermo Fisher Scientific, MA), as per the manufacturer’s guidelines. Residual DNA was removed by DNase I digestion using a TurboDNA-free kit (Thermo Fisher Scientific, MA). cDNA was synthesized using 1 µg RNA, an oligo (dT)_20_ primer and Superscript III RT (Invitrogen, CA). Gene expression of SmAP was measured by quantitative real time PCR (RT-qPCR), using custom TaqMan gene expression systems from Applied Biosystems, CA using primers and probes, as previously (23, 27). Alpha tubulin was used as the endogenous control gene for relative quantification, as described (28) employing the ΔΔCt method (29). Results obtained from parasites treated with the irrelevant, control siRNA were used for calibration. For graphical representation, 2^-ΔΔCt^ values were normalized to controls and expressed as percent difference.

To look for any impact of gene suppression on the ability of worms to cleave vitamin B6, PLP was added to living worms in assay buffer seven days post siRNA treatment and free phosphate levels were measured at selected time points following the protocol described above.

### Cloning cDNAs Encoding the Vitamin B6 Metabolizing Enzymes of Schistosomes

To look for homologs in schistosomes of the human enzymes that are central to vitamin B6 metabolism within cells, we used human sequences [GenBank accession number NP_003672 for pyridoxal kinase (PK, Smp_164250); NP_064711 for Pyridoxal phosphate phosphatase (PLP-Ph, Smp_030220) and NP_060599 for pyridox(am)ine-5’-phosphate oxidase (PNPO, Smp_051550)] to blast against all available “schistosomatidae” family sequences at NCBI (https://blast.ncbi.nlm.nih.gov/Blast.cgi?PAGE=Proteins). Clear sequence homologs were identified in each case and were designated SmPK, SmPLP-Ph and SmPNPO, respectively. Next, primers were generated to target the entire open reading frame of each homolog. These primers were: SmPK**-**F: 5’-ATGGCAGTTAAAGTTGCACTTAAC-3’, SmPK-R: 5’-TCACATTTCTTCAACATAATCACC-3’; SmPLP-Ph-F: 5’-ATGACCAAGGTCGCTTCTGCGGTTCTG-3’, SmPLP-Ph-R: 5’-TTATTCTTTCAAAATATTTAAAATGTCCG-3’; SmPNPO-F: 5’-ATGCTACGAACACTTTACTGTAGCC-3’, SmPNPO-R: 5’-CTATGGAGCTAGTCGTTCGTATACCC-3’. The primers were used in separate PCRs, with adult worm cDNA as template, to amplify the complete schistosome coding sequences. All PCR products were purified, cloned into pCRII-TOPO and transformed into TOP10 *E. coli* (Thermo Fisher Scientific, MA), using standard techniques. Plasmid was purified from a selection of recombinant transformants and several plasmid DNA inserts were sequenced at Genewiz, Inc. MA. The gene encoding SmPK was identified by BLAST analysis using the predicted coding DNA to interrogate the *S. mansoni* genome and NCBI databases.

Phylogenetic trees (absolute distance) were generated for each of the three schistosome proteins and homologs from a variety of organisms by UPGMA (unweighted pair group method with arithmetic mean) using Accelrys Gene 2.5 software.

### SmPK, SmPLP-Ph, and SmPNPO Gene Expression Analysis

The levels of expression of the SmPK, SmPLP-Ph and SmPNPO genes in different life stages of the parasite was measured by reverse transcription quantitative PCR (RT-qPCR), using a custom TaqMan gene expression system (Applied Biosystems). RNA was isolated from different stages of the parasites using TRIzol reagent and cDNA was synthesized as described above. The levels of expression of these genes in different life stages was measured by RT-qPCR using the housekeeping gene triose phosphate isomerase as the endogenous control (27). Primer sets and reporter probes labeled with 6-carboxyfluorescein (FAM), obtained from Applied Biosystems were used. To monitor gene expression, the following primers and probe were used: SmPK-F: 5’-TTGAAAATGAAAACGGACAACAAATAGCT-3’, SmPK-R: 5’-GATAGAACAGTTTGGATTGTACTAAGAACTGA-3’, SmPK probe: 5’-FAM-TTCCTTCAAAGATGCCTTCC-3’; SmPLP-Ph-F: 5’-GTTGCGAATTGGACCCTTCAAAA-3’, SmPLP-Ph-R: 5’-CCAAATTTATTCCCAAATGCGATATCAGT-3’, SmPLP-Ph-probe: 5’-FAM-CTGTGATGGTTGGAGATAACTTAT-3’; SmPNPO-F: 5’-GAAAATGAATATGCTTCAAAAGAAAAGCTTCC-3’, SmPNPO-R: 5’-CCAAAACTCAATTGATTTCGGAGACA-3’, SmPNPO-probe: 5’FAM-TCCCCAATTTGAAGGCTTT-3’. Each RT-qPCR was performed using a TaqMan PCR mix, cDNA and primer and probes in a final volume of 20 µl. All samples were run in triplicate and underwent 40 amplification cycles on a Step One Plus Real Time PCR System Instrument. For relative quantification, the ΔΔCt method was employed.

### Statistical Analysis

Data are presented as Mean ± SD. Means were compared by: t-test (two-tailed, unpaired) for comparison of two groups; one-way ANOVA for comparison of more than two groups using GraphPad Prism 8.0 (GraphPad Software). For metabolite comparisons, Welch’s two-sample t-test was used to identify biochemicals biomolecules that differed significantly between groups. A probability value (P) of less than 0.05 was considered significant.

## Results

### Schistosomes Cleave Exogenous Pyridoxal Phosphate (PLP)

**Figure 1:**
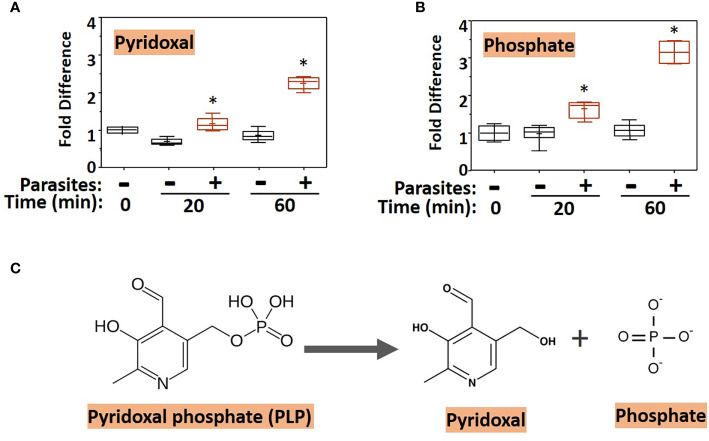
Box plots showing relative levels of pyridoxal **(A)** and phosphate **(B)** in murine plasma that either contained adult schistosomes (+, red) or did not contain schistosomes (-, black) for the indicated time periods. * indicates statistically significant difference compared to the same time point lacking parasites; Welch’s two-sample t-test, P < 0.05. Each box bounds the upper and lower quartile, the line in each box is the median value and “+” signifies the mean value for the sample; error bars indicate the maximum (upper) and minimum (lower) distribution. Values obtained at zero time (0) are set at 1. **(C)** Chemical structure of pyridoxal phosphate (PLP, left) and the products of its hydrolysis (indicated by the arrow), pyridoxal and phosphate (right).

To examine changes brought about by schistosomes on host plasma, adult worms were first incubated in murine plasma as described earlier. Twenty and 60 min later, samples were collected and changes to the plasma metabolome were measured. **Figure 1** illustrates relative changes to pyridoxal (A) and phosphate (B) levels in murine plasma which contained (+) or did not contain (-) adult schistosomes. **Figure 1A** shows that there is a significant increase in the level of pyridoxal at both the 20- and the 60-min time points in the plasma sample that contained worms as well as significant increases in the plasma phosphate level (**Figure 1B**) (P <0.05 in each case). One explanation for these results is that the worms cleave pyridoxal phosphate (PLP, a compound that was not itself monitored in the plasma metabolome) to generate free pyridoxal and phosphate, a reaction that is illustrated in **Figure 1C**. Note that the fold increase in phosphate is markedly greater than that of pyridoxal, suggesting that sources other than PLP account for much of the accumulated phosphate.

**Figure 2:**
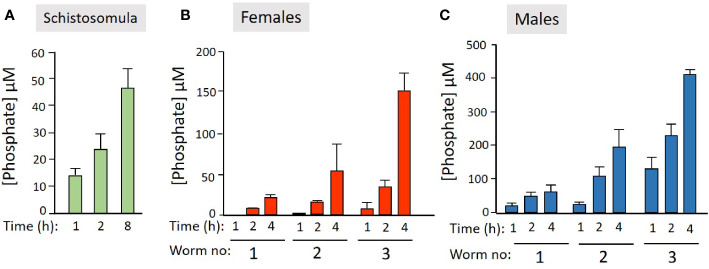
Live schistosome parasites hydrolyze PLP. Phosphate generation (µM, mean +/-SD) by live schistosomula (groups of 2,000, green bars, n = 4, **A**) or adult females (red bars, n = 5, **B**) or adult males (blue bars, n = 5, **C**) in the presence of pyridoxal phosphate (PLP) at the indicated time points. Adult worms were either incubated singly or in groups of two or three, as indicated.

We next examined the ability of live worms to hydrolyze commercially obtained PLP. **Figure 2** shows that all intravascular life stages [schistosomula (**Figure 2A**), adult females (**Figure 2B**), and adult males (**Figure 2C**)] can indeed cleave PLP to release phosphate (which accumulates over time). Schistosomula were tested in groups of ~2,000 and adult worms were tested either singly or in groups of two or three (as indicated). More worms (and longer time points) lead to greater phosphate accumulation.

### Recombinant SmAP can Hydrolyze PLP

**Figure 3:**
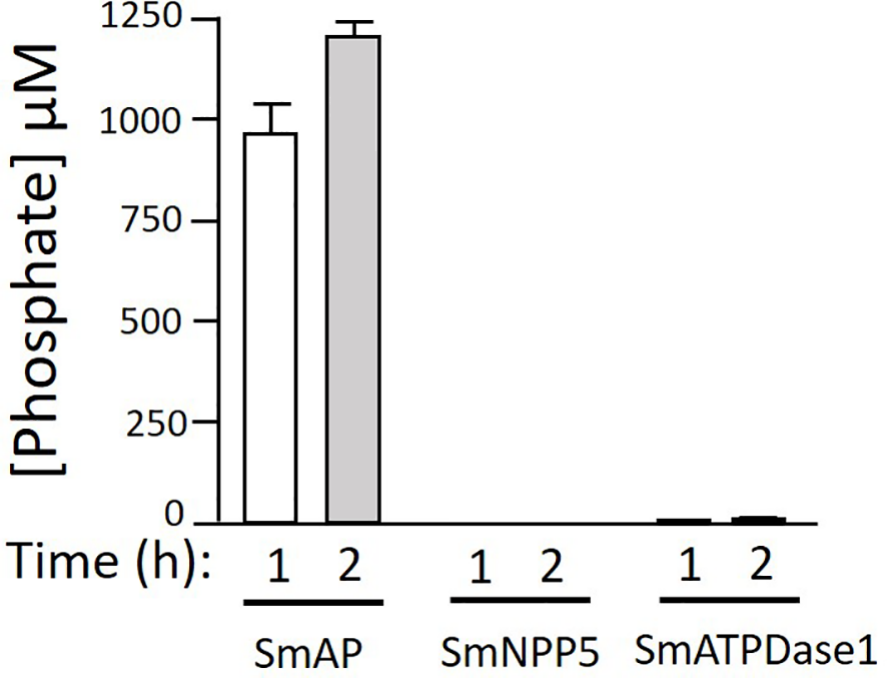
rSmAP can hydrolyze PLP. Phosphate generation (µM, mean +/-SD) by recombinant SmAP, SmNPP5, or SmATPDase1 in assay buffer containing PLP as substrate, at the indicated time points. No appreciable levels of phosphate were detected when rSmNPP5 or rSmATPDase1 were tested.

Next, we tested the ability of three *S. mansoni* ectoenzymes to cleave PLP. These enzymes are: alkaline phosphatase (SmAP), nucleotide phosphodiesterase/pyrophosphatase (SmNPP5), and ATP diphosphohydrolase1 (SmATPDase1); all three have been generated as recombinants, in enzymatically active form in our laboratory (17, 21, 23). **Figure 3** shows that only rSmAP can cleave PLP to liberate phosphate. Neither rSmNPP5 nor rSmATPDase1 are active in this assay [though they can cleave other substrates (17, 30)]. In addition, heat-inactivated rSmAP can no longer cleave PLP (data not shown).

### Tegumental SmAP Cleaves PLP

**Figure 4:**
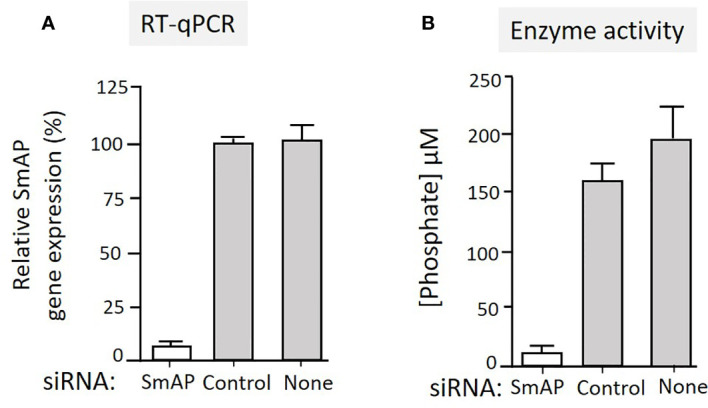
**(A)** Relative SmAP gene expression determined by RT-qPCR in adult male worms 48 h after treatment with an siRNA targeting the SmAP gene or with an irrelevant siRNA (Control) or with no siRNA (None), as indicated. The expression level displayed by the control worms is set at 100%. **(B)** Phosphate generation (µM, mean +/-SD, n = 5) by individual live adult male worms seven days after treatment with SmAP siRNA or an irrelevant siRNA (Control) or no siRNA (None) in the presence of PLP. Parasites treated with SmAP siRNA generate significantly less phosphate compared to either control (One-way ANOVA, P < 0.001).

To test the hypothesis that tegumental SmAP on living worms is responsible for PLP cleavage, adult worms were first treated with siRNAs targeting SmAP or with control siRNAs or with no siRNA. Two days later the relative expression level of the target SmAP gene was compared in all groups. **Figure 4A** shows significant SmAP gene suppression in worms treated with siRNA targeting this gene compared to the controls; the SmAP gene is >90% suppressed as determined by RT-qPCR (p < 0.01). Seven days post siRNA treatment the ability of all worms to hydrolyze PLP was compared and **Figure 4B** shows the results of this experiment. It is clear that living worms whose SmAP gene has been suppressed cleave significantly less PLP compared to control worms treated either with the control siRNA or with no siRNA (P < 0.001). Having less SmAP severely impairs the ability of the living worms to hydrolyze exogenous PLP.

### *In Silico* Analysis of Vitamin B6 Metabolizing Enzymes of *S. mansoni*

**Figure 5:**
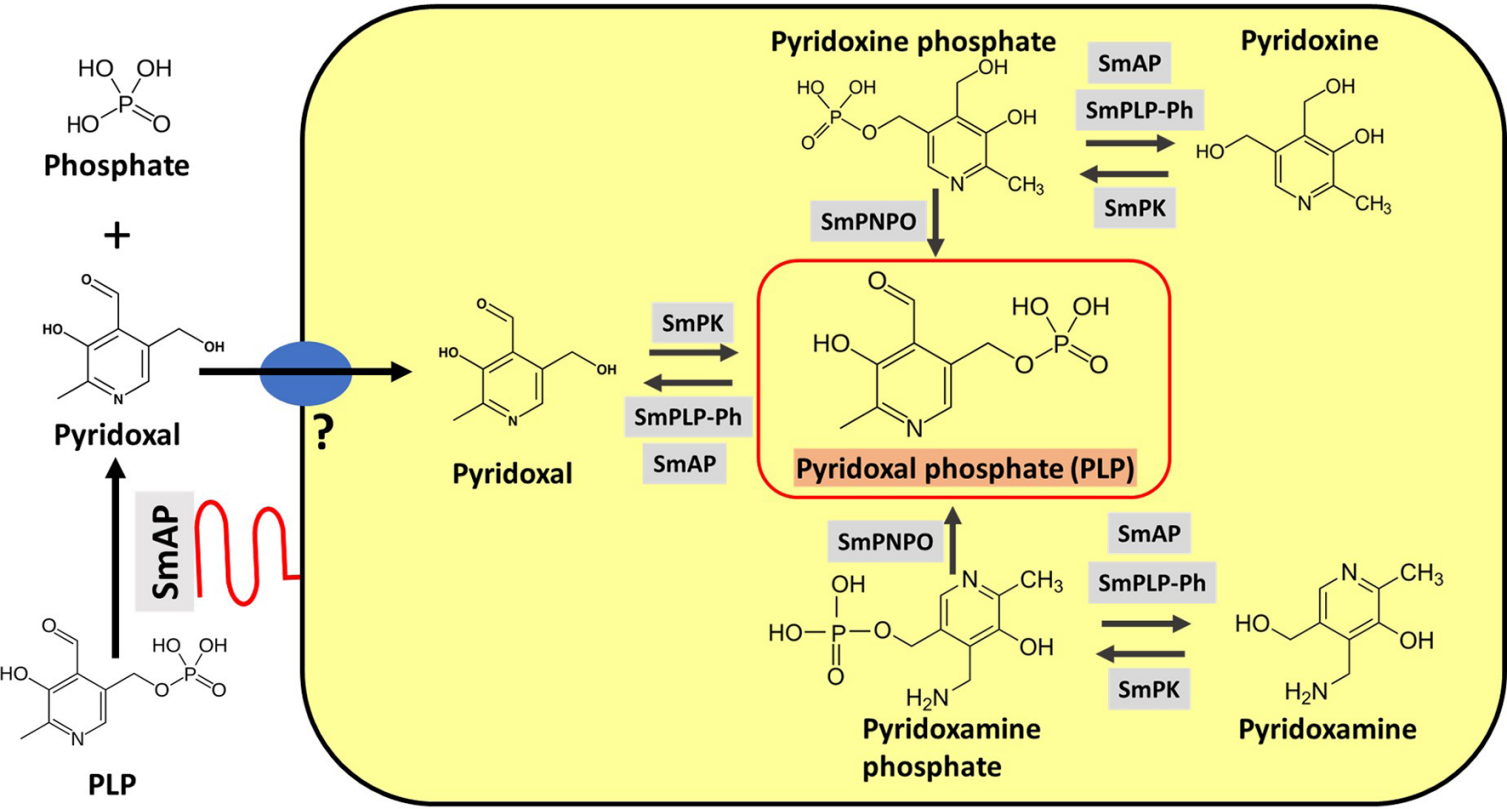
Vitamin B6 metabolism in schistosomes. SmAP (depicted at lower left, red) can cleave PLP to generate free pyridoxal which may be imported by schistosomes *via* an unidentified transporter protein (blue oval, left). Depicted in the yellow rectangle are hypothetical biochemical pathways in *S. mansoni* leading to the generation of PLP (bounded by the red box, center). Imported pyridoxal (left) may be phosphorylated by *S. mansoni* pyridoxal kinase (SmPK) to directly generate PLP; the vitamers pyridoxine (top) and pyridoxamine (bottom) may first be phosphorylated by SmPK to generate pyridoxine phosphate and pyridoxamine phosphate, respectively and these products may then be acted upon by *S. mansoni* pyridox(am)ine-5’-phosphate oxidase (SmPNPO) to generate PLP. Dephosphorylation of several vitamers may be driven by *S. mansoni* pyridoxal phosphate phosphatase (SmPLP-Ph) and/or SmAP.

**Figure 5** depicts SmAP (lower left, red) at the schistosome surface dephosphorylating PLP to generate free pyridoxal which we hypothesize can then be taken into the worms *via* an unidentified transporter protein (**Figure 5**, blue oval). The large yellow rectangle in **Figure 5** contains depictions of hypothesized vitamin B6 metabolic reactions within the parasites as determined by *in silico* analysis, described next.

**Figure 6:**
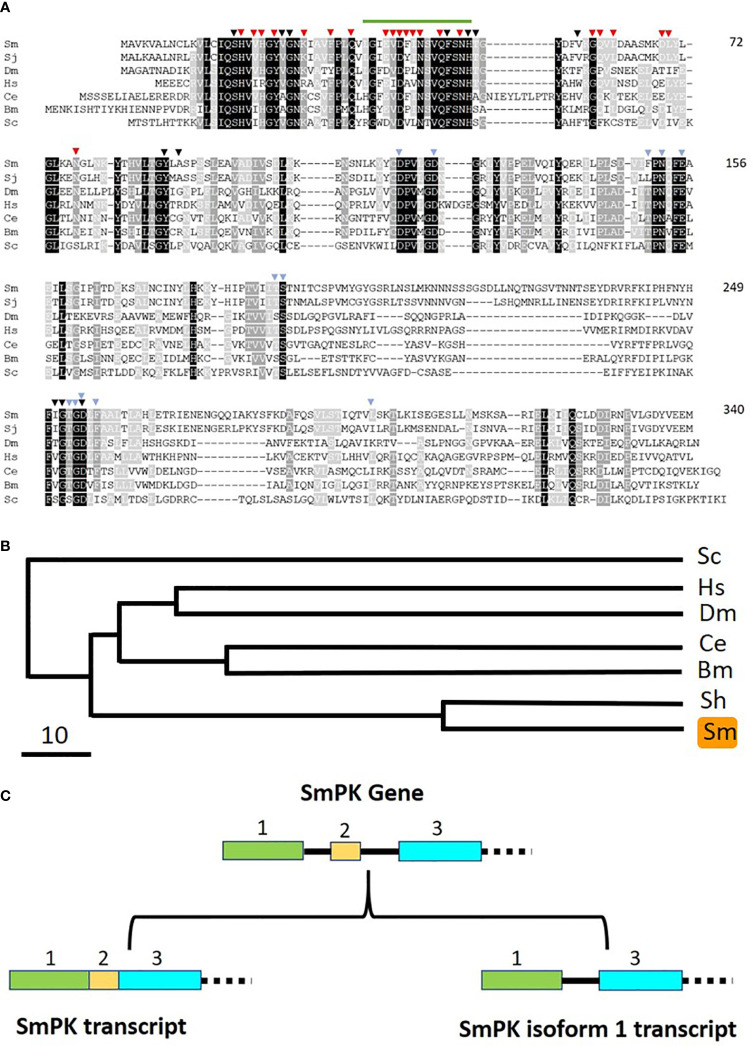
Alignment of SmPK with other members of the pyridoxal kinase protein family. Sm, *Schistosoma mansoni* (GeneBank accession number MW148599); Sj, *Schistosoma japonicum* (CAX69335.1); Hs, *Homo sapiens* (NP_003672); Dm, *Drosophila melanogaster* (AAR82765); Ce, *Caenorhabditis elegans* (NP_491463.2); Bm, *Brugia malayi* (VI094344); Sc, *Saccharomyces cerevisiae* (NP_014424). Residues found in all seven sequences are depicted as white on a black background; those common to six of the seven pyridoxal kinase homologs are white on a dark grey background and those found in a majority of the sequences (four or five out of seven) are white on a light grey background. Residues predicted to be involved in pyridoxal binding are indicated by black arrowheads; those involved in ATP binding by blue arrowheads and those involved in dimer formation, red arrowheads. The green line (top) indicates the sequence motif ^36^LGIEVDFINSVQFSNH^51^ that is replaced by ^36^CVLLIITY^43^ in the SmPK isoform 1 sequence reported in this work (GeneBank accession number of SmPK isoform 1: MW148600). Numbers at right are an amino acid count for the *S. mansoni* protein. **(B)** Phylogenetic tree (absolute distance) of selected pyridoxal kinases generated by multiple sequence alignment using UPGMA (unweighted pair group method with arithmetic mean). The designations (and accession numbers) of the homologs compared are as described in **(A)**. **(C)** Depiction of the 5’end of the SmPK gene (top) and its spliced variants (bottom). In the upper panel, exons 1–3 of the SmPK gene are depicted as colored rectangles (and introns as black lines). In one transcript (lower left panel), both intron 1 and 2 are spliced out. Alternative splicing removes intron 1 and exon 2 but maintains intron 2 to generate the mature SmPK isoform 1 transcript. The dashed line signifies the remainder of the SmPK gene which is identical between transcripts.

If free pyridoxal is taken in by schistosomes, it would need to be phosphorylated internally to regenerate PLP (the active form of vitamin B6). Phosphorylating pyridoxal is a function that is provided in other organisms by pyridoxal kinase (PK) enzymes. Blasting the human PK protein sequence against schistosome sequence databases identified a clear *S. mansoni* homolog. As detailed in Methods, the complete *S. mansoni* pyridoxal kinase (SmPK) coding sequence was amplified by PCR. The purified PCR product was then cloned, and plasmid was isolated from several clones that were sequenced. The full length schistosome SmPK sequence (accession number MW148599) potentially encodes a 340 amino acid protein with a predicted molecular mass of 37,773 daltons and a pI of 5.99. **Figure 6** shows an alignment of this SmPK protein with its counterpart from *S. japonicum* as well as homologs from a variety of other organisms. Residues conserved in all seven sequences are depicted as white on a black background; those common to six of the seven pyridoxal kinase homologs are white on a dark grey background and those found in a majority of the sequences (four or five out of seven) are white on a light grey background. SmPK exhibits several highly conserved domains throughout the protein, including a series of residues that are important for pyridoxal binding (indicated by black arrowheads), residues important for ATP binding (blue arrowheads) and residues key for homodimer formation (red arrowheads) (31).

A phylogenetic tree illustrating the relationships between all PK homologs is depicted in **Figure 6B**. Clearly, the two schistosome sequences are the most closely related and the homolog from the yeast *Saccharomyces cerevisiae* is most distant. Eleven SmPK cloned DNAs were sequenced and, of these, six were found to encode the protein shown in **Figure 6A**. The remaining five SmPK clones all encoded a variant sequence (isoform 1, accession number MW148600) in which the predicted motif ^36^LGIEVDFINSVQFSNH^51^ (indicated by a green line at the top of **Figure 6A**) is replaced by the sequence: ^36^CVLLIITY^43^. This change is brought about by alternative splicing. The **Figure 6C** upper panel illustrates the 5’end of the SmPK gene and shows three of the gene’s 10 exons. The lower panel illustrates two spliced mRNA transcripts. The SmPK transcript is depicted at left; here the first three exons encode part of the mature transcript. The alternatively spliced SmPK isoform 1 transcript is shown at right; here exon 2 is spliced out along with intron 1, while the 31 bp intron 2 remains as part of the mature isoform 1 transcript.

**Figure 7:**
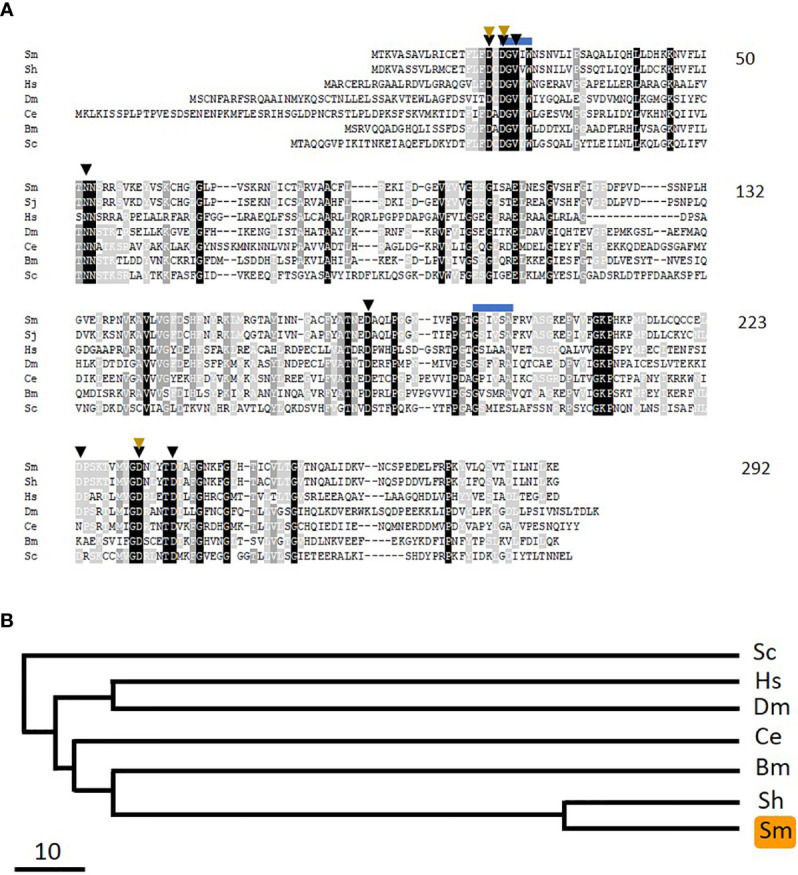
Alignment of SmPLP-Ph with other members of the pyridoxal phosphate phosphatase protein family. Sm, *Schistosoma mansoni* (GeneBank accession number MW148602); Sh, *Schistosoma haematobium* (CAX69944); Hs, *Homo sapiens* (NP_064711); Dm, *Drosophila melanogaster* (NP_649015.2); Ce, *Caenorhabditis elegans* (NP_504511); Bm, *Brugia malayi* (XP_001895356); Sc, *Saccharomyces cerevisiae* (AJV03114). Residue shading is as described in **Figure 6**. Predicted active site residues binding are indicated by black arrowheads and those involved in metal (Mg) binding by brown arrowheads. Blue lines indicate potential myristoylation sites. Numbers at right are an amino acid count for the *S. mansoni* protein. **(B)** Phylogenetic tree (absolute distance) of selected pyridoxal phosphate phosphatases generated by multiple sequence alignment using UPGMA (unweighted pair group method with arithmetic mean). The designations (and accession numbers) of the homologs compared are as in **(A)**.

The reverse reaction to that catalyzed by pyridoxal kinase is commonly driven by PLP-phosphatase (PLP-Ph) enzymes. To determine if schistosomes possess enzymes belonging to this protein class, essentially the same strategy as used to identify SmPK (described above) was applied. Here, the human PLP-phosphatase sequence was used to query schistosome sequence databases. A homolog was identified, and its cDNA was cloned and sequenced (accession number MW148602). The 292 predicted amino acid protein, designated SmPLP-Ph has a MW of 31,915 and a pI of 6.70. **Figure 7A** shows an alignment of the predicted SmPLP-Ph protein with homologs from a variety of other organisms and a phylogenetic tree illustrating the relationships between these is depicted in **Figure 7B**. Conserved motifs are seen throughout the protein with predicted key active site residues indicated by black arrowheads, metal (Mg) binding residues indicated by brown arrowheads and potential myristoylation sites indicated by blue lines (32, 33).

**Figure 8:**
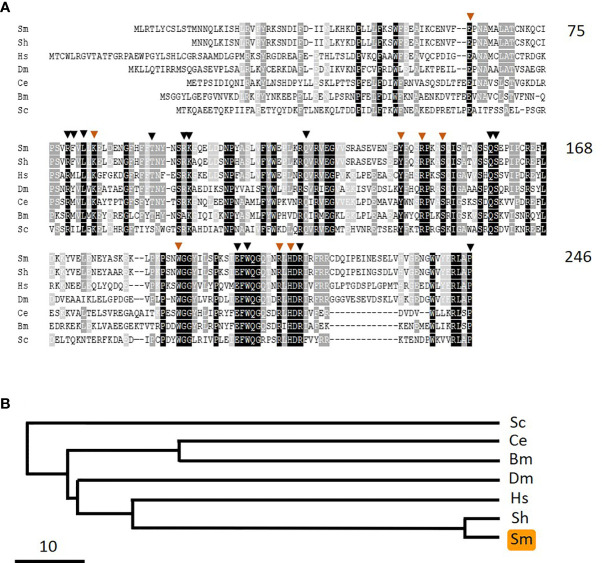
Alignment of SmPNPO with other members of the pyridox(am)ine 5’-phosphate oxidase protein family. Sm, *Schistosoma mansoni* (GeneBank accession number MW148601); Sh, *Schistosoma haematobium* (XP_012792194); Hs, *Homo sapiens* (NP_060599); Dm, *Drosophila melanogaster* (NP_731186.2); Ce, *Caenorhabditis elegans* (NP_498518); Bm, *Brugia malayi* (XP_001896760); Sc, *Saccharomyces cerevisiae* (AJQ03502). Residue shading is as described in **Figure 6**. Residues predicted to be important in pyridoxal phosphate binding are indicated by brown arrowheads and those involved in binding the cofactor flavin mononucleotide are indicated by black arrowheads. Numbers at right are an amino acid count for the *S. mansoni* protein. **(B)** Phylogenetic tree (absolute distance) of selected pyridox(am)ine 5’-phosphate oxidases generated by multiple sequence alignment using UPGMA (unweighted pair group method with arithmetic mean). The designations (and accession numbers) of the homologs compared are as in **(A)**.

As noted earlier, pyridoxine and pyridoxamine are vitamin B6 vitamers. In the same way that pyridoxal is phosphorylated to generate PLP, likewise pyridoxine and pyridoxamine can each be phosphorylated by pyridoxal kinase to generate pyridoxine phosphate and pyridoxamine phosphate, respectively. An oxidase enzyme [pyridox(am)ine 5’-phosphate oxidase, PNPO] is then required to convert these compounds into PLP—the biologically active form of vitamin B6. In order to assess whether schistosomes possess a PNPO homolog (SmPLPO) we applied the same strategy that was used successfully to find SmPK and SmPLP-Ph. Here, the human PNPO sequence was used to query schistosome sequence databases and a homolog was identified. The cDNA encoding this match was amplified, cloned and its insert sequenced (accession number MW148601). The predicted SmPNPO sequence is shown in **Figure 8A** aligned with several homologs and a phylogenetic tree illustrating the relationships between these is depicted in **Figure 8B**. The 246 amino acid SmPNPO protein has a predicted molecular mass of 28,878 daltons and a pI of 6.55 and exhibits several highly conserved domains including a series of residues important for PLP binding (orange arrowheads) as well as those used to bind co-factor flavin mononucleotide (FMN, black arrowheads) (34).

Phylogenetic trees chart the relationships between the three *S. mansoni* vitamin B6 metabolizing enzymes SmPK (**Figure 6B**), SmPLP-Ph (**Figure 7B**), and SmPNPO (**Figure 8B**) and their closest homologs from the same seven other organisms. Overall, the SmPK tree, the SmPLP-Ph tree and the SmPNPO tree are similar—e.g., the proteins of the two schistosome species are most closely related while that from the yeast *Saccharomyces cerevisiae* is consistently most distant. However, even though the proteins likely have the same metabolic function involving vitamin B6 biochemistry in all of the organisms, the three trees are not identical. This likely reflects the diverse selection pressures exerted on the proteins during the evolutionary histories of each of the different organisms.

Not surprisingly, several genes encoding proteins that require PLP have been annotated in the *S. mansoni* genome including aspartate aminotransferase (Smp_064380), ornithine-oxo-acid transaminase (Smp_000660), 5-aminolevulinic acid synthase (Smp_045260), serine C-palmitoyltransferase (Smp_028080), and glycogen phosphorylase (Smp_143840). This highlights the importance of PLP for widely diverse aspects of schistosome biochemistry.

### Developmental Expression of SmPK, SmPLP-Ph, and SmPNPO

**Figure 9:**
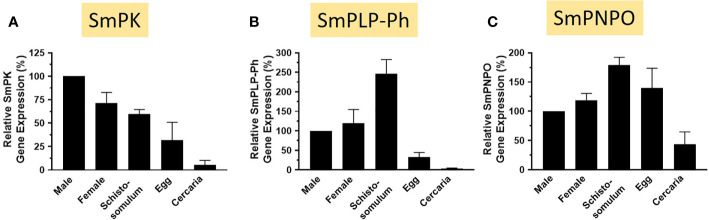
Expression of SmPK, SmPLP-Ph and SmPNPO in different *S. mansoni* life stages. Relative gene expression of SmPK **(A)**, SmPLP-Ph **(B)** and SmPNPO **(C)** in the life stages indicated. All values are relative to males (set at 100%) (Mean +/- SD, n = 3).

The relative expression levels of SmPK, SmPLP-Ph, and SmPNPO, assessed by RT-qPCR, are depicted in **Figures 9A–C**. In all cases the expression level in adult males is set at 100%. For each of the three genes, lowest relative expression is seen in the cercarial life stage and robust expression is seen in all intravascular life stages (adults and schistosomula). SmPLP-Ph exhibits notably high relative expression in schistosomula and expression of SmPNPO (**Figure 9C**) differs from that of SmPK and SmPLP-Ph by having high relative expression also in the egg stage.

## Discussion

When schistosomes are incubated in murine plasma they alter its metabolomic profile; the levels of two of the metabolites measured (pyridoxal and phosphate) increase over time. This change is most easily understood if the worms have an ability to cleave any pyridoxal phosphate (PLP) that is available in the plasma thereby generating free pyridoxal and phosphate. Since intact PLP was not included in the panel of metabolites monitored, we do not see here a commensurate drop in PLP levels that would be predicted to accompany the observed increase in pyridoxal and phosphate. PLP is the active form of vitamin B6 and plays critical roles in cellular biochemistry. Animals, including schistosomes, are auxotrophic for vitamin B6 and require it as a supplement. The intravascular worms must acquire this vital metabolite from their hosts. These factors inspired us to investigate how the worms might obtain and metabolize vitamin B6.

We first show here that all intravascular schistosome life stages tested (schistosomula as well as adult males and females) can cleave exogenous PLP. Since PLP hydrolysis was mediated by live, intact worms this suggests that the enzyme responsible is expressed at the schistosome surface.

We have long studied the molecular biology of the host-interactive schistosome tegument and have identified several key host-interactive proteins (35–37). Among the characterized proteins are three ectoenzymes that are strong candidates for mediating the cleavage of PLP by the worms, as seen in this work. All three are nucleotide metabolizing ectoenzymes (NMEEs) and include an ATPdiphosphohydrolase1 (SmATPDase1), a phosphodiesterase/pyrophosphatase (SmNPP5) and an alkaline phosphatase, (SmAP) (17, 21, 23). Current known substrates for SmATPDase1 are ATP and ADP (17); for SmNPP-5, ADP, and NAD (21, 30), and for SmAP: AMP, GMP, CMP, TMP, S1P, and polyP (23, 24). Here we explored the ability of each of these three ectoenzymes, all produced in active recombinant form in CHO cells, to cleave PLP. Of the three, only SmAP was seen to hydrolyze PLP and this ability was lost upon enzyme heat-inactivation. Furthermore, parasites whose SmAP gene was suppressed by RNAi were significantly impaired in their ability to cleave PLP compared to controls. This proves that SmAP present at the surface of intravascular schistosomes can cleave exogenous PLP. The very substantial decrease (> 90%) in PLP cleavage observed in the SmAP-suppressed worms strongly suggests that SmAP is the sole schistosome ectoenzyme with this capability.

Much of the literature on schistosome ectoenzyme function focuses on the ability of the enzymes to maintain an anti-inflammatory and anti-thrombotic environment around the worms (38). ATP is a pro-inflammatory, damage-associated molecular pattern (DAMP) that may be cleaved by SmATPDase1 (17). This enzyme, as well as SmNPP5, can cleave the pro-coagulant, platelet-activator ADP (17, 21). SmAP cleaves the pro-thrombotic, immune modulators S1P and polyP (23, 24). SmAP additionally cleaves AMP to generate the potently anti-inflammatory mediator, adenosine (22). In addition to its proposed role in impairing host purinergic signaling [by cleaving AMP to generate the signaling molecule adenosine (38)], SmAP may also have a role in parasite feeding; its action may generate metabolites in a form that permits their easy transportation into the body of the worm. SmAP-dephosphorylation of not just AMP but also of GMP, CMP, and TMP [to generate adenosine, guanosine, cytosine, and thymidine, respectively (23)] may facilitate the efficient uptake of these nucleosides into the worms as food—an important function since schistosomes cannot synthesize purines *de novo* (39). We argue that, in a comparable manner, SmAP-mediated cleavage of membrane-impermeable PLP will generate free pyridoxal in the vicinity of the parasites from where it can be easily imported into the body of the worms. In similar fashion, in vertebrates, the absorption of PLP first involves dephosphorylation catalyzed by a membrane-bound alkaline phosphatase (11, 40).

Precisely how any pyridoxal that is generated following PLP cleavage is taken into any cell remains unclear; whether by passive diffusion and/or by carrier-mediated absorption (11, 41). In either case, uptake is driven by the intracellular trapping of the vitamin *via* the formation of 5′-phosphates through the action of a cytosolic ATP-dependent pyridoxal kinase (11). To explore the possibility that schistosomes possess such an enzyme that could likewise trap any imported pyridoxal, we queried schistosome sequence databases for the presence of homologs of the human PK. In this manner, a 340 amino acid schistosome protein—SmPK belonging to the PK protein family was identified that possesses strongly conserved motifs known to be involved in pyridoxal and ATP binding. Our analysis also revealed that alternative splicing can lead to the generation of a 332 amino acid SmPK variant (isoform 1) which lacks several conserved residues at its amino terminal end. How this deletion might impact the dimerization ability or other functions of SmPK is unclear and remains to be investigated.

PLP within cells may be dephosphorylated to generate pyridoxal which can then be taken up by other cells within the body and in this manner vitamin B6 vitamers can become distributed between tissues (10, 11). In schistosomes, SmAP could fulfill this PLP-dephosphorylation function; the enzyme is widely distributed in schistosomes (18, 22) and we have shown here that it can cleave PLP to generate free pyridoxal. However, since SmAP is a GPI-linked ectoenzyme, whether it is available to contribute to intracellular, cytosolic PLP metabolism is called into question. In other systems, a phosphatase more dedicated to this task—PLP-Ph cleaves PLP intracellularly and we show here that schistosomes possess a clear PLP-Ph homolog (SmPLP-Ph) with several highly conserved motifs characteristic of this protein family.

A third enzyme that is key to vitamin B6 metabolism is a pyridox(am)ine-5’-phosphate oxidase (PNPO). This enzyme converts the vitamin B6 vitamers pyridoxine phosphate and pyridoxamine phosphate into the active form of the vitamin—pyridoxal phosphate. Again, examination of schistosome sequence databases reveals a clear schistosome PNPO homolog (SmPNPO) which contains multiple conserved motifs throughout the predicted protein that are characteristic of this protein family.

Taken together our analysis suggests that if schistosomes take in any B6 vitamer (pyridoxine, pyridoxamine, or pyridoxal) from the blood, they possess the enzymes necessary to convert these into the active form of the vitamin—PLP. The pathways involved are illustrated in **Figure 5**, yellow rectangle. SmPK could convert pyridoxal directly to PLP. SmPK could phosphorylate both pyridoxine (top right, **Figure 5**) and pyridoxamine (bottom right, **Figure 5**) to generate pyridoxine phosphate and pyridoxamine phosphate, respectively and these two metabolites could be converted to PLP *via* SmPNPO. Finally, the phosphatase SmPLP-Ph, and/or SmAP, could dephosphorylate several vitamers to permit easier movement of B6 metabolites between tissues.

Developmental expression analysis of SmPK, SmPLP-Ph, and SmPNPO reveals high relative expression of all three in intravascular schistosome life stages, suggestive of robust vitamin B6 metabolism within the final host. The expression patterns of the genes encoding each of these enzymes are distinct, likely reflecting differences in the biochemical pathways that are most engaged in the various life stages.

The work presented here extends the known substrates upon which SmAP can act; in addition to AMP, GMP, CMP, TMP, S1P, and polyP, PLP is now added to the list. We hypothesize that SmAP at the surface of intravascular schistosomes plays a key role in vitamin B6 acquisition by the parasites. The protein can cleave PLP in the blood to generate a pool of free pyridoxal in the vicinity of the worms from where this vitamer might conveniently be taken up. Phosphorylation of pyridoxal *via* SmPK would trap the vitamer inside the worms as PLP which could then engage in the many biochemical reactions that require this co-factor.

It has been reported that plasma PLP gets mobilized to sites of inflammation for use by PLP‐dependent enzymes that play active roles in inflammatory processes e.g., the PLP‐dependent reactions that are necessary for immune cell proliferation or to generate immunomodulatory sphingolipids (42). Thus, it is possible that SmAP-mediated cleavage of plasma PLP not only generates free pyridoxal as a nutrient for the worms but additionally deprives the host of sufficient PLP for optimal inflammation. Furthermore, pyridoxal alone has been shown capable of inhibiting production of inflammatory cytokines in macrophages (43). Thus, it is possible that any pyridoxal generated by the action of SmAP that is not imported into the parasites could contribute to a biochemical anti-inflammatory perimeter established by the worms around themselves.

Blocking SmAP function in schistosomes might impair parasite feeding and metabolism by diminishing the available pool of pyridoxal around the worms (in addition to impeding the parasite’s ability to generate the anti-inflammatory mediators adenosine and pyridoxal). Thus, new therapies that inhibit SmAP might block both parasite feeding and immunomodulation and could help to promote optimal inflammatory responses thereby controlling these globally debilitating pathogens.

## Data Availability Statement

The datasets presented in this study can be found in online repositories. The name of the repository and accession numbers can be found here: https://www.ncbi.nlm.nih.gov/genbank/, MW148599, MW148600, MW148602, MW148601.

## Ethics Statement

The animal study was reviewed and approved by Tufts University IACUC.

## Author Contribution

AD: experiment design and data collection, analysis and interpretation, writing. ME: data collection, analysis and interpretation, critical review. SE-B: supervision, critical review. PS: project concept and design, data analysis, supervision, writing. All authors contributed to the article and approved the submitted version.

## Funding

This work was funded with support from NIH-NIAID grant AI056273. Infected snails were provided by BRI *via* the NIAID schistosomiasis resource center under NIH-NIAID Contract No. HHSN272201000005I.

## Conflict of Interest

The authors declare that the research was conducted in the absence of any commercial or financial relationships that could be construed as a potential conflict of interest.

## Abbreviations

PL, pyridoxal; PN, pyridoxine; PM, pyridoxamine; PLP, pyridoxal phosphate; SmPK, *Schistosoma mansoni* pyridoxal kinase; SmPNPO, *Schistosoma mansoni* pyridox(am)ine 5’-phosphate oxidase; SmPLP-Ph, *Schistosoma mansoni* pyridoxal phosphate phosphatase.
